# A comparative study of the svm and k-nn machine learning algorithms for the diagnosis of respiratory pathologies using pulmonary acoustic signals

**DOI:** 10.1186/1471-2105-15-223

**Published:** 2014-06-27

**Authors:** Rajkumar Palaniappan, Kenneth Sundaraj, Sebastian Sundaraj

**Affiliations:** 1 AI-Rehab Research Group, Kampus Pauh Putra, Universiti Malaysia Perlis (UniMAP), Perlis, Malaysia; 2 Department of Anesthesiology, Klang General Hospital, Klang, Malaysia

**Keywords:** Respiratory sounds, MFCC, One way ANOVA, Support vector machine, K-nearest neighbour, Confusion matrix

## Abstract

**Background:**

Pulmonary acoustic parameters extracted from recorded respiratory sounds provide valuable information for the detection of respiratory pathologies. The automated analysis of pulmonary acoustic signals can serve as a differential diagnosis tool for medical professionals, a learning tool for medical students, and a self-management tool for patients. In this context, we intend to evaluate and compare the performance of the support vector machine (SVM) and K-nearest neighbour (K-nn) classifiers in diagnosis respiratory pathologies using respiratory sounds from R.A.L.E database.

**Results:**

The pulmonary acoustic signals used in this study were obtained from the R.A.L.E lung sound database. The pulmonary acoustic signals were manually categorised into three different groups, namely normal, airway obstruction pathology, and parenchymal pathology. The mel-frequency cepstral coefficient (MFCC) features were extracted from the pre-processed pulmonary acoustic signals. The MFCC features were analysed by one-way ANOVA and then fed separately into the SVM and K-nn classifiers. The performances of the classifiers were analysed using the confusion matrix technique. The statistical analysis of the MFCC features using one-way ANOVA showed that the extracted MFCC features are significantly different (*p* < 0.001). The classification accuracies of the SVM and K-nn classifiers were found to be 92.19% and 98.26%, respectively.

**Conclusion:**

Although the data used to train and test the classifiers are limited, the classification accuracies found are satisfactory. The K-nn classifier was better than the SVM classifier for the discrimination of pulmonary acoustic signals from pathological and normal subjects obtained from the RALE database.

## Background

Auscultation is the process of listening to the internal sounds of the body using a stethoscope. This process provides vital information on the present state of the internal organs, such as the heart and lungs [1]. The stethoscope, which was invented by René Théophile Hyacinth Laennec in 1816, has been used to perform auscultation for several years. The auscultation process is inexpensive, non-invasive, and less time-consuming [2]. Computer-based respiratory sound analysis started to appear in the literature in the early 1980s. This method can assist medical professionals with differential diagnoses, which are used to diagnose the specific disease suffered by a patient or to at least eliminate any imminent life-threatening conditions. The sensors that are most commonly used for computerised respiratory sound recording are microphones, accelerometers, and digital stethoscopes. The types and characteristics of the respiratory sounds that are widely accepted have been reported by Pasterkamp *et al*. [3]. The normal respiratory sound dominant frequency ranges from 37.5 to 1000 Hz. The dominant frequency of airway obstruction pathology is less than 400 Hz, and the dominant frequency of parenchymal pathology ranges from 200 to 2000 Hz. The duration of airway obstruction pathological conditions, such as wheeze and rhonchi, is greater than 250 ms, whereas the duration of parenchymal pathological conditions, such as crackles, is less than 100 ms. These respiratory sound characteristics provided by Pasterkamp *et al*. clearly introduced the possibility of discriminating respiratory sounds using signal processing algorithms. However, further studies are required before computerised respiratory sound analysis can be implemented in a clinical setting. In particularly, the development of a robust system requires the implementation of more sophisticated signal processing and machine learning algorithms.

### Related works on respiratory sound analysis

Previous studies on computerised respiratory sound analysis have been conducted using various signal processing and machine learning algorithms [4]. This section provides a discussion of the few recent prominent works on computerised respiratory sound analysis. In the study conducted by Güler *et al*. [5], normal, wheeze, and crackles respiratory sounds were classified using their power spectral density features. Electret microphones were used to record the respiratory sounds from 129 subjects, and these were then classified using artificial neural networks (ANNs) and genetic algorithm (GA)-based ANNs. The classification accuracies found for ANN and GA-based ANN were 81-91% and 83-93%, respectively. Alsmadi *et al*. [6] proposed the use of an autoregressive model for the classification of respiratory sounds. These researchers used an ECM-77B microphone to record the respiratory sounds from 42 subjects and then implemented the k-nearest neighbour algorithm (k-nn) to classify the respiratory sounds. The recognition rate was found to be 96%. Dokur *et al*. [7] proposed an incremental supervised neural network for the classification of respiratory sounds. These researchers used ECM-77B Electret microphones to acquire the respiratory sounds from 18 subjects and then extracted the power spectrum features of the respiratory sounds. They then used a grow-and-learn (GAL) network, which is an incremental supervised neural network, for the classification of the respiratory sounds and found that their classification accuracy was promising compared with the previously proposed methods. Sankar *et al*. [8] proposed a feedforward neural network for the classification of normal and pathological respiratory sounds based on the following features: energy index, respiration rate, dominant frequency, and strength of the dominant frequency. These researchers used an Electret microphone to record the respiratory sounds from six subjects and obtained a classification accuracy of 98.7%. In the same year, Hashemi *et al*. [9] proposed the use of wavelet-based features for the classification of respiratory sounds using a multi-layer perceptron network. These researchers used an electronic stethoscope to record the respiratory sounds from 140 subjects, and their experimental results show that their system can achieve a recognition rate of 89.28%. Flietstra *et al*. [10] used support vector machine for the recognition of respiratory sounds. These researchers used an STG 16 lung sound analyser to record the respiratory sounds from 257 subjects and the statistical median feature to train and test the SVM classifier. A mean classification accuracy of 84% was reported.

Even though there are studies in this field dating back to the early 1980s, computerised respiratory sound analysis has not yet been implemented to a level that can be used in a clinical setting. The advancements in signal processing in recent years allow us to use more sophisticated methods for respiratory sound analysis. The literature review revealed that both the feature extraction method and the machine learning algorithm play major roles in the recognition of respiratory sounds. This study compares two different approaches for the recognition of the respiratory pathologies using pulmonary acoustic signals. More specifically, the support vector machine (SVM) and k-nearest neighbour (k-nn) classifiers were implemented for the differentiation of normal, airway obstructions pathology, and parenchymal pathology conditions using the cepstral features obtained from respiratory sounds in the RALE database.

### Methodology

**Figure 1:**
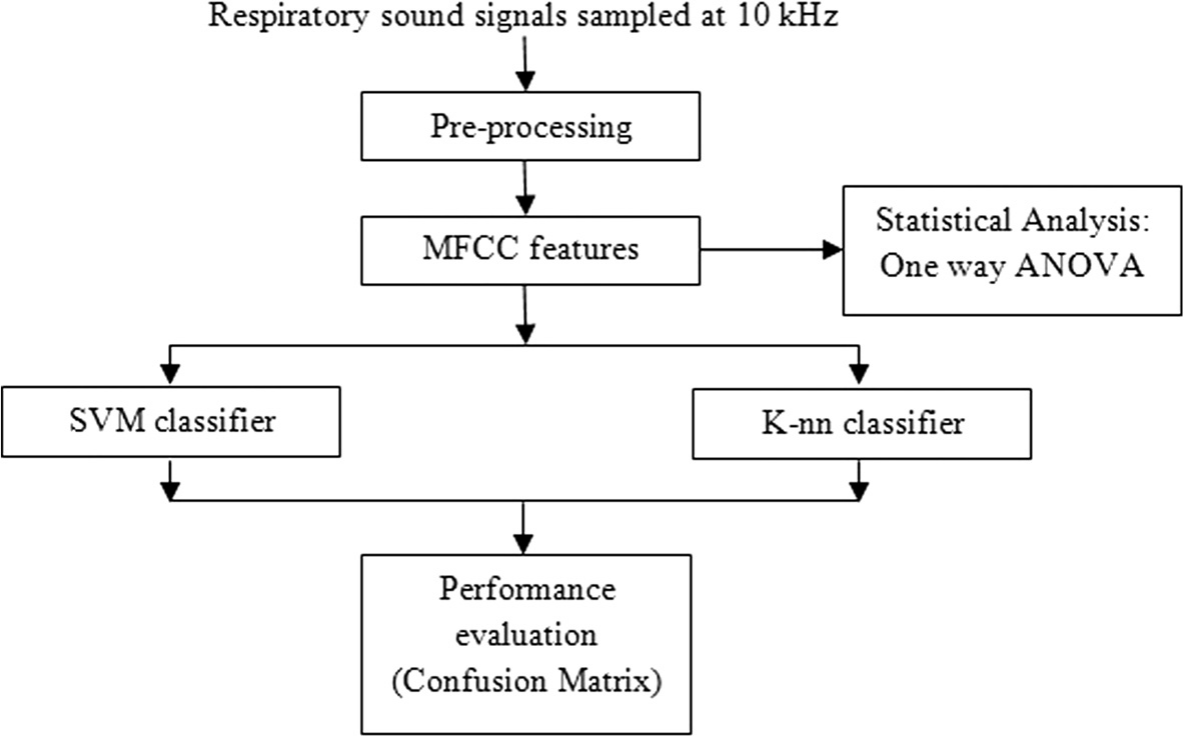
Block diagram of pulmonary acoustic signal processing.

The respiratory sounds used in this study were obtained from the RALE database, which is the only commercially available respiratory sound database and has been used by many researchers. The proposed system includes four processing stages, namely preprocessing, feature extraction, classification, and performance evaluation. In the feature extraction stage, the MFCC features are extracted from the respiratory sound signals, and these are fed to the SVM and k-nn classifiers separately in the classification stage. The SVM and k-nn classifiers were used to distinguish normal, airway obstruction, and parenchymal pathologies. A block diagram of the proposed work is illustrated in Figure 1.

### Respiratory sound database

**Figure 2:**
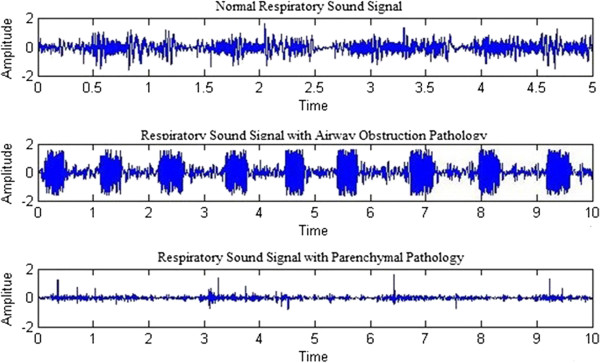
Signal plot of respiratory sounds associated with normal and pathological conditions.

The respiratory sounds that were used in this work were obtained from the R.A.L.E database, which is the only commercially available respiratory sound database [4]. The R.A.L.E database comprises of more than 70 recordings from various subjects that were recorded on the surface of the chest wall using a contact accelerometer (EMT25C, Siemens). These recordings were manually categorised into three different groups, namely normal pathology, airway obstruction pathology, and parenchymal pathology. Previous studies on respiratory sound analysis states that here is only negligible change in frequency intensity when the age progresses. Even the gender difference in respiratory sound analysis was of no difference. It is not necessary to consider the age difference or the gender difference in pulmonary acoustic signal analysis [11,12]. A total of 68 recordings were obtained from the database,the remaining 2 data were collected from infants and were not considered for this study. Of the 68 recordings that were considered in this research work, 17 were indicative of normal pathology, 26 were associated with airway obstruction pathology, and 25 were related with parenchymal pathology. Figure 2 shows the respiratory sound signals associated with normal, airway obstruction, and parenchymal pathologies obtained from the R.A.L.E database.

### Respiratory sound Pre-processing

Respiratory sound signals are subject to noise, such as heart sound and other artefacts [1]. The RALE database comprises recordings that have been filtered to remove the heart sounds and artefacts. The respiratory sound signals were high-pass filtered at 7.5 Hz to remove the DC offset using a first-order Butterworth filter and low-pass filtered at 2.5 kHz to avoid aliasing using an eight-order Butterworth filter. The sampling rate of the respiratory sounds was 10 kHz [13].

### Parametric representation

**Figure 3:**
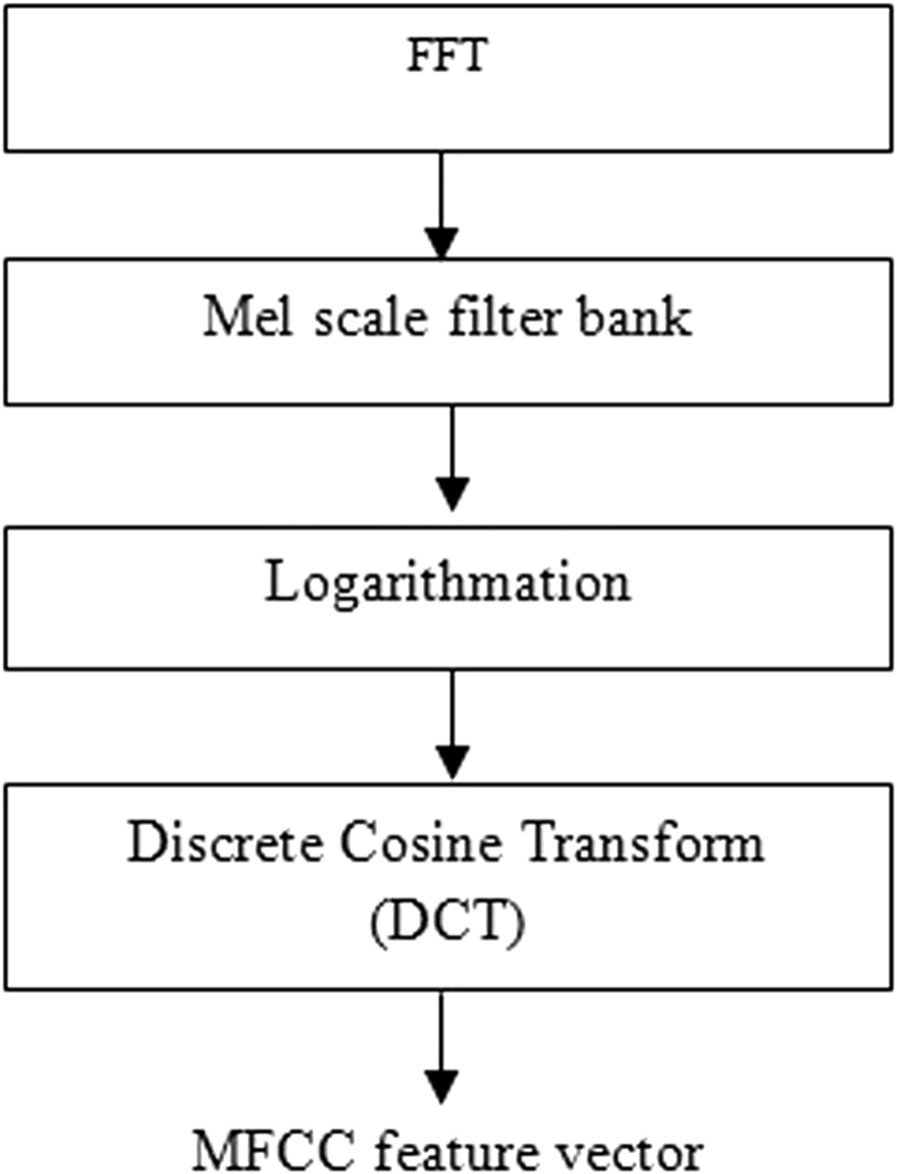
Block diagram of the process used to obtain the MFCC feature vector.

The spectral characteristics of the pulmonary acoustic signals are vital in determining the respiratory pathology [14]. One of the parametric representation which depicts the spectral characteristics of signals is Mel-frequency cepstral coefficients (MFCC). MFCC are associated with a highly effective feature extraction algorithm used in speech signal processing. Furthermore, some researchers have previously implemented MFCC for respiratory sound analysis and have obtained promising outcomes [15]. MFCC analysis is similar to cepstral analysis apart from frequency wrapping. In MFCC analysis, the frequency is wrapped in accordance with the mel scale, which more closely approximates the human auditory system’s response. In contrast, cepstral analysis involves linearly spaced frequency bands using a normal cepstrum [16]. Figure 3 shows the step-by-step process used to obtain the MFCCs. The mel-frequency cepstral coefficients are calculated from the Fast Fourier Transform (FFT) coefficients, which are filtered using a triangular bandpass filter bank known as the mel scale filter bank. The linear frequency is mapped to the mel-frequency using Eq. (1):

(1) $$\mathrm{Mel}\left(f\right)=2595\log_{10}\left(1+\frac{f}{700}\right)$$

where Mel(f) is the logarithmic scale of the normal frequency scale f. The logarithmic scale is then converted to time through the use of a discrete cosine transform, and the output is the set of MFCCs. The MFCCs obtained from the respiratory sounds are then used as features in the SVM and k-nn classifiers. In this study, 13 MFCCs were extracted for the classification of the respiratory sounds.

### Statistical test

In this study, analysis of variance (ANOVA) was used to test the significance of the feature vector. One-way ANOVA is used to test the null hypothesis of samples with more than two groups. More specifically, one-way ANOVA is used to test the equality of three or more means at one time using the variances [17].

### Classification

In this work, two different classifiers were used, namely support vector machine (SVM) and k-nearest neighbour (k-nn). A detailed description of the classifiers used can be found in this section.

### Support Vector Machine (SVM)

The SVM classifier is a kernel-based supervised learning algorithm that classifies the data into two or more classes. SVM is particularly designed for binary classification. During the training phase, SVM builds a model, maps the decision boundary for each class, and specifies the hyperplane that separates the different classes. Increasing the distance between the classes by increasing the hyperplane margin helps increase the classification accuracy. SVM can be used to effectively perform non-linear classification. Detailed information on the SVM classifier can be found in [18,19]. In this study, the MFCC feature vector was fed to the SVM classifier to distinguish normal, airway obstruction, and parenchymal pathological conditions. As mentioned earlier, the SVM classifier is a kernel based classifier. A Kernel function is a mapping procedure done to the training set to improve its resemblance to a linearly separable data set. The purpose of mapping is to increase the dimensionality of the data set and it is done efficiently using a kernel function. Some of the commonly used kernel functions are linear, RBF, quadratic, Multilayer Perceptron kernel, and Polynomial kernel. In this research work, linear and RBF kernel functions were used. The linear kernel function and RBF kernel functions were used due to their dissimilar characteristics. The linear kernel function performs well with linearly separable data set and the RBF kernel function performs well with non-linear data set. The linear kernel function takes less time to train the SVM compared to the RBF kernel function. The linear kernel function is less prone to over fitting compared to the RBF kernel function [20,21]. The performance of the SVM classifier relies on the choice of the regularization parameter C which is also known as box constraint and the kernel parameter which is also known as the scaling factor. Together they are known as the hyperplane parameter. The value of the box constraint C for the soft margin was set to 1 for both linear and RBF kernel. The scaling factor σ for the RBF kernel was set to 1.

### K-Nearest Neighbor (k-nn)

In pattern recognition, the k-nn algorithm is instance based learning method used to classify objects based on their closest training examples in the feature space. An object is classified by a majority vote of its neighbours, i.e., the object is assigned to the class that is most common amongst its k-nearest neighbours, where k is a positive integer [22]. In the k-nn algorithm, the classification of a new test feature vector is determined by the classes of its k-nearest neighbours. Here, the k-nn algorithm was implemented using Euclidean distance metrics to locate the nearest neighbour [23]. The Euclidean distance metrics d(x, y) between two points x and y is calculated using the Eq. (2). Where N is the number of features such that x = {x_1_,x_2_,x_3_…x_N_} and y = {y_1_,y_2_,y_3_…y_N_}. The number of neighbours (i.e., k) used to classify the new test vector was varied in the range of 1 to 10, and its effects on the classification performance were determined in the form of classification accuracy with standard deviation.

(2) $$d\left(x,y\right)=\sum_{i=1}^{N}\sqrt{{x_{i}}^{2}-{y_{i}}^{2}}$$

## Results and discussion

The extracted MFCC features were tested using one-way ANOVA, and the features were found to be significantly different between the groups (F (2, 68) = 4397.1, *p* < 0.001). The MFCC features were separately binary normalised and bipolar normalised. The normalization is done to standardize the range of independent variables. Normalization also improves the effectiveness and the performance of the machine learning algorithms [24]. In this work, two validation schemes, namely conventional validation and ten-fold cross-validation, were used to verify the reliability of the outcome of the classifier. In the conventional validation scheme, the data were partitioned into two sets, namely the training and the testing set. The training set comprises 60% of the data, and the remaining 40% of the data formed the testing set. The classifier was trained and tested 25 times through the conventional validation scheme using randomly assigned training and testing sets, and the classification accuracy and standard deviation are reported. In the ten-fold cross-validation scheme, the dataset was divided randomly into 10 sets of size n/10, where n is the total number of datapoints. The training was conducted using nine sets, and the remaining set was used for testing. This method was repeated 10 times, and the average mean classification accuracy is reported. The performance outcome of the SVM and k-nn classifiers for binary-normalised features are reported in Tables 1 and 2, respectively. The performance outcome of the SVM and k-nn classifiers for bipolar-normalised features are reported in Tables 3 and 4, respectively. The averages and standard deviations of the classification accuracies are tabulated. The standard deviation of the classification clearly reveals the consistency of the classifier results. If the standard deviation is higher, the classification results are considered inconsistent, and this inconsistency also depends on the parameters of the classifier.

**Table 1:** Performance outcome of the SVM classifier for binary-normalised data

**Kernel**	**Validation method**	**Classification accuracy (%)**
Linear	Conventional validation	86.91 ± 1.47
RBF		89.54 ± 0.39
Linear	Ten-fold cross-validation	90.13 ± 0.54
RBF		91.47 ± 1.66

**Table 2:** Performance outcome of the k-nn classifier for binary-normalised data

**K value**	**Validation method**	**Classification accuracy (%)**	**Validation method**	**Classification accuracy (%)**
1	Conventional validation	94.66 ± 0.56	Ten-fold cross-validation	96.65 ± 0.69
2		94.81 ± 0.42		96.35 ± 0.93
3		93.92 ± 0.68		95.06 ± 0.34
4		94.53 ± 0.47		94.41 ± 0.62
5		94.25 ± 0.94		95.02 ± 1.21
6		93.95 ± 1.15		94.41 ± 1.35
7		92.70 ± 1.27		93.20 ± 1.08
8		92.57 ± 0.85		94.44 ± 1.28
9		92.49 ± 0.63		93.32 ± 0.74
10		91.77 ± 0.77		92.17 ± 1.46

**Table 3:** Performance outcome of the SVM Classifier for bipolar-normalised data

**Kernel**	**Validation method**	**Classification accuracy (%)**
Linear	Conventional validation	89.17 ± 1.32
RBF		91.36 ± 1.69
Linear	Ten-fold cross-validation	89.91 ± 2.39
RBF		92.19 ± 1.58

**Table 4:** Performance outcome of the k-nn Classifier for bipolar-normalised data

**K value**	**Validation method**	**Classification accuracy (%)**	**Validation method**	**Classification accuracy (%)**
1	Conventional validation	97.53 ± 0.29	Ten-fold-cross validation	98.26 ± 0.32
2		97.62 ± 0.58		97.11 ± 0.75
3		97.56 ± 1.25		97.52 ± 0.97
4		96. 23 ± 1.36		97.67 ± 0.46
5		96.92 ± 2.54		97.53 ± 0.48
6		95.25 ± 1.27		97.82 ± 0.62
7		95.51 ± 1.58		96.65 ± 0.96
8		95.88 ± 1.69		96.41 ± 1.19
9		95.88 ± 0.58		96.65 ± 1.36
10		95.14 ± 1.69		96.25 ± 0.85

As shown in Table 1, the SVM classifier with the RBF kernel obtained the maximum classification accuracy of 89.54% with a standard deviation of 0.39 for the binary-normalised data with the conventional validation method. Similarly, as shown in Table 2, the k-nn classifier with a k value of 2 gave the maximum classification accuracy of 94.81% with a standard deviation of 0.42 for the binary normalised data with the conventional method. Table 3 shows that the SVM classifier with the RBF kernel gives the maximum classification accuracy of 91.36% with a standard deviation of 1.69 for the bipolar-normalised data with the conventional validation method. Similarly, as shown in Table 4, the k-nn classifier with a k value of 2 obtained the maximum classification accuracy of 97.62% with a standard deviation of 0.58 for the bipolar-normalised data with the conventional validation method.

The data shown in Table 1 reveal that the SVM classifier with the RBF kernel gives the maximum classification accuracy of 91.47% with a standard deviation of 1.66 for the binary-normalised data with the ten-fold cross-validation method. Similarly, Table 2 shows that the k-nn classifier with a k value of 1 gives the maximum classification accuracy of 96.65% with a standard deviation of 0.69 for the binary-normalised data with the ten-fold cross-validation method. Table 3 reveals that the SVM classifier with the RBF kernel obtains the maximum classification accuracy of 92.19% with a standard deviation of 1.58 for the bipolar-normalised with the ten-fold cross-validation method. Similarly, as shown in Table 4, the k-nn classifier with a k value of 1 gives the maximum classification accuracy of 98.26% with a standard deviation of 0.32 for the bipolar-normalised data with the ten-fold cross-validation method. The results obtained demonstrate that the k-nn classifier outperform the SVM classifier in the discrimination of respiratory pathologies. The classification accuracies show that the SVM classifier with the RBF kernel and the ten-fold cross-validation method yields the maximum classification accuracy for the diagnosis of respiratory pathology. Similarly, the k-nn classifier with a k value of 1 and the ten-fold cross-validation method yields the maximum classification accuracy. Both of these machine learning methods achieve the maximum classification accuracy when the data are bipolar normalised.

The approximation in the classification accuracies are due to the multiple trials conducted using random training and testing set. In the case of conventional validation scheme the machine learning algorithm (SVM and KNN) were trained and validated for 25 times (trials). Each of these 25 trials has random samples for training and testing data which results in the approximation in accuracy. Similarly for the 10 fold cross validation scheme the models were trained and validated for 10 times. In the ten-fold cross-validation scheme, the dataset were divided randomly into 10 sets of size n/10, where n is the total number of data points. The training was conducted using nine sets, and the remaining 1 set was used for testing. This method was repeated 10 times by altering/shifting the 1 set used for testing. Tables 5 and 6 depict the evaluation of the performance of the SVM and k-nn classifiers using the confusion matrix technique. In these tables, N refers to the normal data, A is the airway obstruction pathology, and P refers to the data associated with the parenchymal pathology. The results obtained using the confusion matrix (Table 5) show that a normal respiratory sound was misclassified as parenchymal pathology in one instance. In addition, an airway obstruction pathology was misclassified as a normal respiratory sound in one instance, and a parenchymal pathology was misclassified as normal in two instances and as an airway obstruction pathology in one instance. Similarly, the results obtained using the confusion matrix shown in Table 6 demonstrate that a parenchymal pathology was misclassified as airway obstruction pathology in one instance.

**Table 5:** Confusion matrix for the SVM classifier (Best results: kernel = rbf; normalisation = bipolar)

	**Predicted**				
		N	A	P	Accuracy
	N	16	0	1	94.12%
**Actual**	A	1	25	0	96.13%
	P	2	1	22	88.00%

**Table 6:** Confusion matrix for the k-nn classifier (best results: k value = 1; Normalisation = bipolar)

	**Predicted**				
		N	A	P	Accuracy
	N	17	0	0	100%
**Actual**	A	0	26	0	100%
	P	0	1	24	96%

This comparative study shows that the generalisation capability of the k-nn classifier is higher compared with that of the SVM classifier in the diagnosis of respiratory pathologies from the RALE database. However, the computational complexity of the k-nn classifier is high compared to SVM classifier [25]. The results obtained using the k-nn classifier shows that when the k value is less, the classifier performs better. If we have a dataset with n datapoints, then the n-nearest neighbor classifier will always use all datapoints in the dataset to classify new points, since the k-nearest neighbor classifiers uses a majority voting scheme. In view of this when k = 1, only the nearest one data point is chosen. Increasing the k value increases the number of neighbors which may lead to a decrease in performance because the chances of including a data point from a different class becomes higher with the increase of nearest neighbours [26]. The incremental property of the k-nn machine learning algorithm is better than the SVM classifier [25]. This property allows the k-nn classifier to perform better than the SVM classifier in classifying the pulmonary acoustic signals. The pulmonary acoustic signals are non-linear and non-stationary signals [14]. The k-nn classifier is a non-linear classifier and the SVM is both linear and non-linear [25]. When the linear kernel function is used the SVM acts as a linear classifier and when the RBF kernel is used the SVM acts as the non-linear classifier. The classification accuracy of the SVM with linear kernel is low compared to other classifiers because of the non-linear and non-stationary properties of the pulmonary acoustic signals. The limitation of this study is the number of data used. The number of data used in this study is very low and the data collection was carried out in a controlled environment. The analysis of data with respect to clinical settings should be carried out in future with a larger database. The analysis can be further extended to other feature extraction techniques and machine learning algorithms.

## Conclusion

This study compared the performance of the SVM and k-nn classifiers for the classification of respiratory pathologies from the RALE lung sound database. To do so, the MFCC features of respiratory sounds obtained from the RALE database were extracted. The extracted feature vectors were analysed through one-way ANOVA and were found to be highly significantly different (*p* < 0.001). The maximum classification accuracies for the SVM and k-nn classifiers were found to be 92.19% and 98.26%, respectively. The maximum classification accuracy of the SVM classifier was obtained with the RBF kernel, the ten-fold cross-validation method, and bipolar-normalised data. Similarly, the maximum classification accuracy of with the k-nn classifier was obtained for a k value of 1, the ten-fold cross-validation method, and bipolar-normalised data. These findings show that the generalisation capability of the k-nn classifier is higher compared with that of SVM for the classification of respiratory pathologies from the RALE lung sound database.

## Competing interests

The authors of this article declare that they have no competing interests.

## Authors’ contributions

RP implemented algorithms, carried out analysis and drafted the manuscript, KS participated in the design of the study and coordination, SS participated in the design of the study and performed the statistical analysis. All authors read and approved the final manuscript.

## References

[B1] Palaniappan R Sundaraj K Ahamed NU Arjunan A Sundaraj S Computer-based respiratory sound analysis: a systematic review IETE Tech Rev 2013 30 248 256 10.4103/0256-4602.113524

[B2] Abbas A Fahim A An automated computerized auscultation and diagnostic system for pulmonary diseases J Med Syst 2010 34 1149 1155 10.1007/s10916-009-9334-1 20703592

[B3] Pasterkamp H Kraman SS Wodicika G Respiratory sounds advances beyond the stethoscope Am J Respir Crit Care Med 1997 156 974 987 10.1164/ajrccm.156.3.9701115 9310022

[B4] Palaniappan R Sundaraj K Ahamed NU Machine learning in lung sound analysis: a systematic review Biocybern Biomed Eng 2013 33 129 135 10.1016/j.bbe.2013.07.001

[B5] Güler İ Polat H Ergün U Combining neural network and genetic algorithm for prediction of lung sounds J Med Syst 2005 29 217 231 10.1007/s10916-005-5182-9 16050077

[B6] Alsmadi S Kahya YP Design of a DSP-based instrument for real-time classification of pulmonary sounds Comput Biol Med 2008 38 53 61 10.1016/j.compbiomed.2007.07.001 17716642

[B7] Dokur Z Respiratory sound classification by using an incremental supervised neural network Pattern Anal Appl 2009 12 309 319 10.1007/s10044-008-0125-y

[B8] Sankar AB Kumar D Seethalakshmi K Neural network based respiratory signal classification using various feed-forward back propagation training algorithms Eur J Sci Res 2011 49 468 483

[B9] Hashemi A Arabalibiek H Agin K Classification of wheeze sounds using wavelets and neural networks International Conference on Biomedical Engineering and Technology 2011 IACSIT Press: IACSIT Press 127 131

[B10] Flietstra B Markuzon N Vyshedskiy A Murphy R Automated analysis of crackles in patients with interstitial pulmonary fibrosis Pulm Med 2011 2011 1 7 10.1155/2011/590506 PMC3115658 21738873

[B11] Gross V Dittmar A Penzel T SchÜTtler F von Wichert P The relationship between normal lung sounds, age, and gender Am J Respir Crit Care Med 2000 162 905 909 10.1164/ajrccm.162.3.9905104 10988103

[B12] Fiz JA Jane’ R Lozano M Go’mez R Ruiz J Detecting unilateral phrenic paralysis by acoustic respiratory analysis PLoS ONE 2014 9 e93595 10.1371/journal.pone.0093595 24718599 PMC3981712

[B13] Pasterkamp H RALE: A computer-assisted instructional package Respir Care 1990 35 1006

[B14] Palaniappan R Sundaraj K Sundaraj S Artificial intelligence techniques used in respiratory sound analysis – a systematic review Biomedizinische Technik/Biomed Eng 2014 59 7 18 10.1515/bmt-2013-0074 24114889

[B15] Bahoura M Pattern recognition methods applied to respiratory sounds classification into normal and wheeze classes Comput Biol Med 2009 39 824 843 10.1016/j.compbiomed.2009.06.011 19631934

[B16] Mayorga P Druzgalski C Morelos RL Gonzalez OH Vidales J Acoustics based assessment of respiratory diseases using GMM classification Annual International Conference of the IEEE Engineering in Medicine and Biology Society (EMBC) 2010 2010 Buenos Aires: IEEE 6312 6316 10.1109/IEMBS.2010.5628092 21097364

[B17] Mahapoonyanont N Mahapoonyanont T Pengkaew N Kamhangkit R Power of the test of one-way Anova after transforming with large sample size data Procedia Soc Behav Sci 2010 9 933 937

[B18] Tsai C-F Hsu Y-F Lin C-Y Lin W-Y Intrusion detection by machine learning: a review Expert Syst Appl 2009 36 11994 12000 10.1016/j.eswa.2009.05.029

[B19] Cortes C Vapnik V Support-vector networks Mach Learn 1995 20 273 297

[B20] Suykens JAK Vandewalle J Least squares support vector machine classifiers Neural Process Lett 1999 9 293 300 10.1023/A:1018628609742

[B21] Maji S Berg AC Malik J Classification using intersection kernel support vector machines is efficient IEEE Conference on Computer Vision and Pattern Recognition 2008 Anchorage, AK: IEEE 1 8

[B22] Hmeidi I Hawashin B El-Qawasmeh E Performance of KNN and SVM classifiers on full word Arabic articles Adv Eng Inform 2008 22 106 111 10.1016/j.aei.2007.12.001

[B23] Pan F Wang B Hu X Perrizo W Comprehensive vertical sample-based KNN/LSVM classification for gene expression analysis J Biomed Inform 2004 37 240 248 10.1016/j.jbi.2004.07.003 15465477

[B24] Quackenbush J Microarray data normalization and transformation Nat Gene 2002 32 496 501 10.1038/ng1032 12454644

[B25] Bhaskar H Hoyle DC Singh S Machine learning in bioinformatics: a brief survey and recommendations for practitioners Comput Biol Med 2006 36 1104 1125 10.1016/j.compbiomed.2005.09.002 16226240

[B26] Beyer K Goldstein J Ramakrishnan R Shaft U Beeri C, Buneman P When is “nearest neighbor” meaningful? Database Theory — ICDT’99 1999 1540 London, UK: Springer-Verlag 217 235 10.1007/3-540-49257-7_15

